# Development of an Overhauser Magnetometer with an Omnidirectional Sensor for Submarine Magnetic Field Observation

**DOI:** 10.3390/s26206666

**Published:** 2026-10-21

**Authors:** Jianxiang Xu, Shuang Zhang, Shudong Chen, Xin Guo, Zhihang Li

**Affiliations:** 1 College of Electronic Science and Engineering, Jilin University, Changchun 130012, China; jxxu22@mails.jlu.edu.cn (J.X.); zhangshuang@jlu.edu.cn (S.Z.); zhihang23@mails.jlu.edu.cn (Z.L.); 2 Changbaishan Laboratory, Jilin University, Changchun 130012, China; 3 College of Physics, Jilin University, Changchun 130012, China

**Keywords:** Overhauser sensor, omnidirectional sensor, marine magnetometer, marine station magnetometer

## Abstract

To achieve optimal measurement of the Earth’s magnetic field at different latitudes when the attitude of the sensor changes, an omnidirectional sensor is necessary for marine magnetometers. A novel omnidirectional sensor consisting of primary and compensation coils is proposed to enhance omnidirectional performance. The primary coil consists of eight inclined coils with an inclined angle of 27° to generate both axial and radial magnetic field components, and two compensation coils are wound to enhance radial magnetic field components. With the compensation coils, the omnidirectional coefficient was successfully increased from 0.411 to 0.485 through theoretical calculations. Experimentally, the measured omnidirectional coefficient was 0.477, which is in agreement with the theoretical result. The performance of the omnidirectional sensor was estimated in a controlled artificial magnetic field. For the worst case, the peak-to-peak amplitudes of the Larmor signal and sensitivity are 1.817 V and 0.018 nT@5s (sensitivity with a cycling rate of 5 s), respectively. For the best case, the peak-to-peak amplitudes of the Larmor signal and sensitivity are 3.808 V and 0.004 nT@5s, respectively.

## 1. Introduction

Marine base station magnetometers, which are deployed under the sea through an anchoring placement method, can continuously observe variations in the Earth’s magnetic field [1]. They not only improve the accuracy of ocean magnetic measurements through diurnal correction but also provide data for optimizing geomagnetic models and conducting ocean and space science [2].

Proton magnetometers, optically pumped magnetometers, and fluxgate magnetometers are widely used in marine magnetic surveys. Traditional proton magnetometers have many advantages, such as a simple structure and good omnidirectionality [3]. However, high power consumption limits their application in long-term unattended seabed observation [4]. Optically pumped magnetometers have the characteristics of high sensitivity and high sampling rates [5]. However, they usually suffer from measurement dead zones and high power consumption, making it difficult to implement long-term observation at different latitudes [6]. Fluxgate magnetometers can realize three-component magnetic field detection but require a three-axis orthogonal structure for omnidirectional measurement, resulting in large volume, low integration, significant temperature drift, and poor long-term stability [7].

The Overhauser magnetometer (OVM), which is based on the Overhauser effect (electron–nuclear spin polarization), has many advantages, such as high sensitivity, high accuracy, low temperature drift, and excellent long-term stability with ultra-low power consumption, so it is suitable for long-term unattended seabed observation [6]. The performance of non-omnidirectional sensors strongly depends on the angle between the Earth’s field and the sensor axis, and this leads to significant differences in the amplitude of sensor signals at different latitudes and attitudes. Since omnidirectionality is crucial for measurement accuracy, optimizing the design of these sensors is of great significance [8,9,10,11]. In addition, ultra-low power consumption is necessary for long-term uninterrupted observation [12]. In summary, omnidirectional measurement capability and low power consumption are important for marine base station magnetometers.

The coils of traditional OVM sensors are mostly solenoid shaped. The DC polarization magnetic field is predominantly concentrated along the sensor’s axial direction [13]. When the direction of the geomagnetic field aligns with the polarization magnetic field, the bias field decreases sharply, resulting in a reduced Larmor signal. This lowers the signal-to-noise ratio, leading to inaccurate magnetic field data and larger errors. Altering the structure of the sensor’s omnidirectional coil and enhancing its omnidirectionality can effectively address this issue. Existing omnidirectional coils include the spherical coil from France [14], but it is too complex and difficult to fabricate. J. Ge proposed a multi-angle solenoid coil, which achieves an omnidirectional rate of 38% [15]. In this paper, we propose a new coil structure that significantly improves the omnidirectional coefficient of the sensor.

The main content of the paper is summarized as follows. Firstly, the basic working principles of the OVM are discussed. Secondly, we introduce the total structure of the marine OVM with an omnidirectional sensor that includes a coaxial resonant and omnidirectional coil. Finally, the amplitude of the FID signal and the sensitivity of the sensor at different angles are evaluated in a low-magnetic measurement laboratory, followed by an assessment of the sensitivity of the marine OVM in a field environment.

## 2. Physical Principles

The working process of an OVM can be divided into three phases: (1) the RF magnetic field generated by radio-frequency polarization causes the electrons in the free-radical solution to produce electron spin resonance (ESR); (2) the polarization is transferred from the excited electron spins to the hydrogen nuclear spins, and this process is called dynamic nuclear polarization (DNP); and (3) the nuclear magnetic resonance generated in a hydrogen-rich solution produces a time-decaying Larmor signal through transverse relaxation.

### 2.1. ESR

When placing a solution of the ^15^N nitrogen–oxygen free radical TEMPONE in an external magnetic field ***B*_0_** _=_ ***B*_0_e_z_**, the time-independent Hamiltonian can be described in angular frequency units as follows [16]:

*H* = −*γ*_S_***B*****_0_ ⋅ S_Z_** − *γ*_K_***B*****_0_ ⋅ K_Z_** + *A***K ⋅ S** (1)

**Figure 1:**
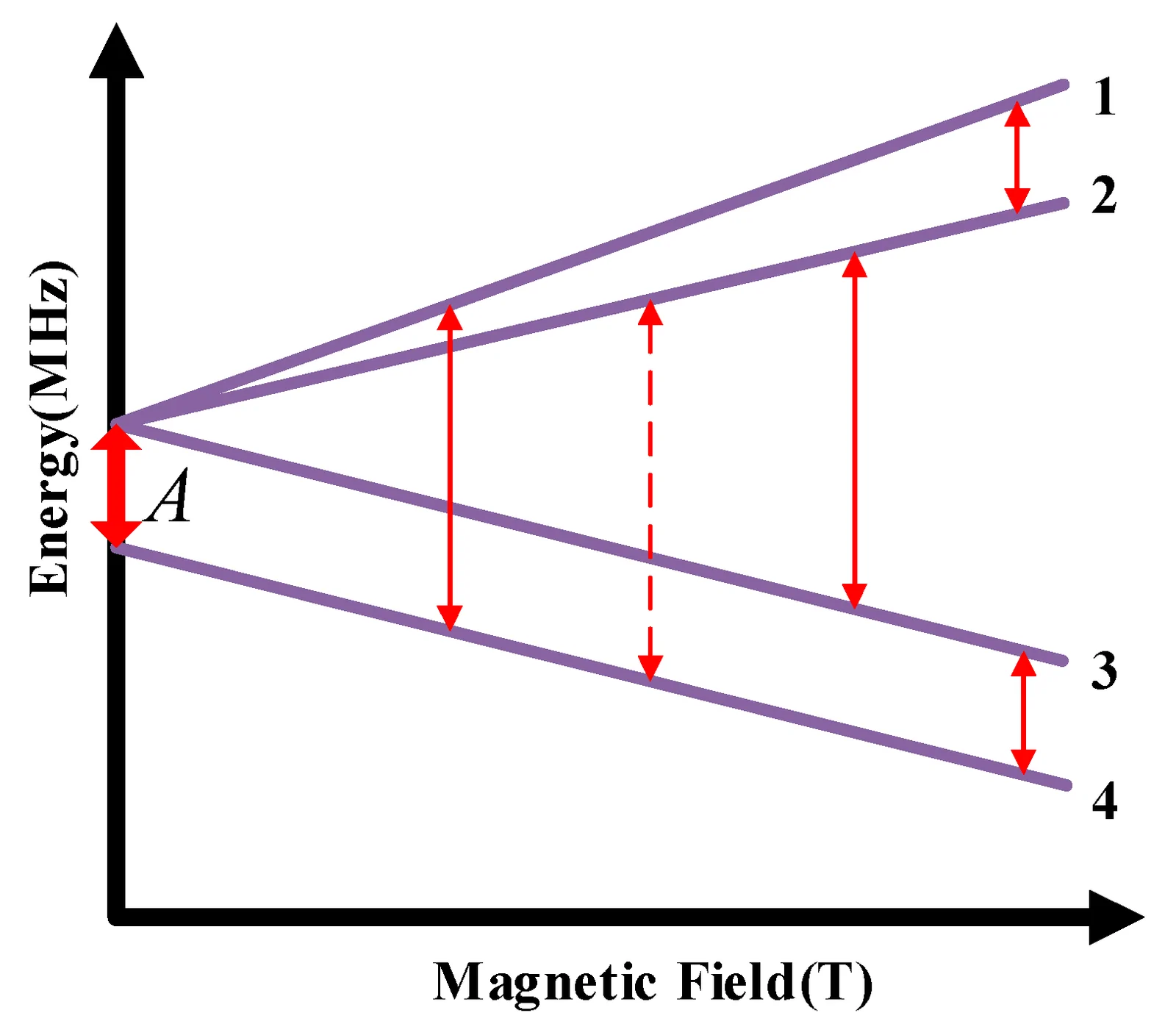
Energy levels of the ^15^N nitroxide radical in the Earth’s field.

Here, S is the electron spin constant, K is the nuclear spin constant, and *γ*_S_ and *γ*_K_ are the gyromagnetic ratios of the electron spin and nuclear spin, respectively. A is the hyperfine coupling constant for the two-spin system. For the ^15^N TEMPONE free-radical solution, S = 1/2 and K = 1/2 [17]. In the ***B*_0_** field, the unpaired electron in the free-radical solution couples with the ^15^N nucleus via hyperfine interaction, and Zeeman splitting occurs in the external magnetic field. According to the Breit–Rabi formula [18], under the influence of RF, the energy levels of the ^15^N TEMPONE free radical split into four levels, as shown in Figure 1. The component of the RF parallel to the external magnetic field ***B*_0_** induces σ transitions, represented by the red dashed line in the energy level diagram; the component of the RF perpendicular to the external magnetic field ***B*_0_** induces *π* transitions, represented by solid red lines.

### 2.2. Dynamic Nuclear Polarization

**Figure 2:**
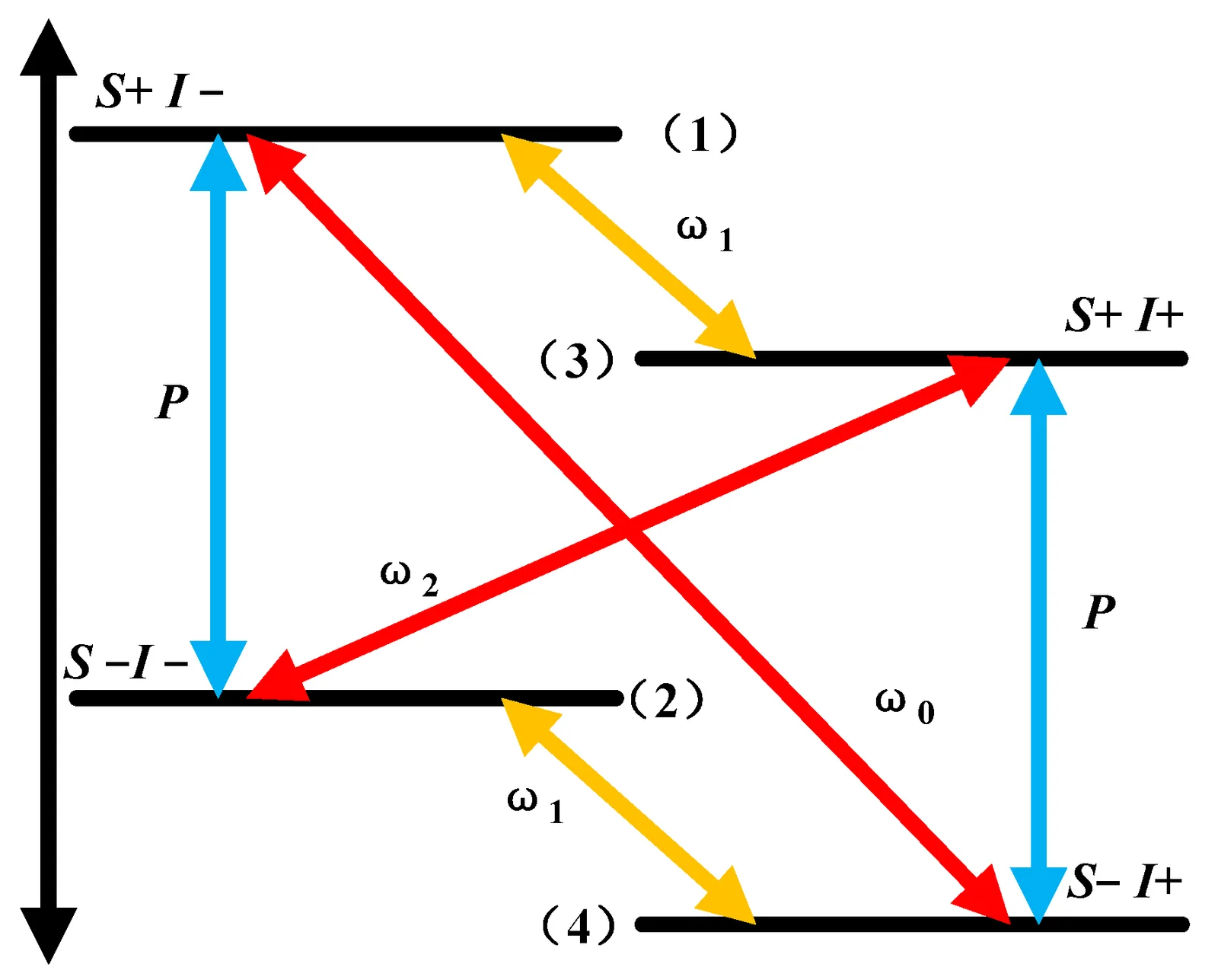
Energy level coupled between proton and electron.

In low fields, DNP in a paramagnetic solution is an effective method for enhancing nuclear magnetic resonance (NMR) signals. This enhancement (Overhauser effect) is achieved through magnetic coupling between the protons and the unpaired electrons in the free radicals. The coupled system has four energy levels, as shown in Figure 2 [19].

*S* and *I* represent the electron spin and proton spin, respectively. *ω*_0_, *ω*_1_, *ω*_2_ and *P* represent the transient probabilities of transitions between the four energy levels. *P* is the transition of the unpaired electron in the free-radical solution from *S*+ to *S*− (or *S*− to *S*+), which reflects the electron spin relaxation rate. *ω*_1_ is the transition of the proton from *I*+ to *I*− (or *I*− to *I*+), which reflects the proton spin relaxation rate. *ω*_0_ and *ω*_2_ are the zero-quantum and double-quantum relaxation rates, representing simultaneous transitions of the electron and proton.

The Overhauser effect occurs when the electron spin relaxation *P* is saturated by a radio-frequency alternating field, driving the electron into a non-equilibrium state. Through zero-quantum relaxation *ω*_0_ or double-quantum relaxation *ω*_2_, this cross-relaxation transfers the electron spin energy to the proton spin energy, thereby enhancing proton spin polarization in the direction of the geomagnetic field. The DNP enhancement factor *F* is given by [20,21]

(2) $$F=\frac{\langle I_{Z}\rangle }{\langle I_{0}\rangle }=1-\rho \mathrm{fs}\frac{\left|\gamma_{S}\right|}{\gamma_{I}}$$

where ‹*I*_Z_› is the expectation value of the dynamic nuclear polarization and ‹*I*_0_› is its thermal equilibrium value. ρ represents the coupling factor; when there is hyperfine interaction between electron spin *S* and nuclear spin *I*, ρ has a value of −1. f is the leakage factor, determining the efficiency of the electron’s influence on nuclear relaxation. In an ideal case, f is essentially 1, meaning that nuclear spin *I* relaxes exclusively through the coupling with electron spin *S*. The saturation factor s measures the degree of electron transition saturation, and when the electron transition is fully saturated, *s* = 1. Therefore, under ideal conditions, the DNP enhancement factor *F* is determined by the ratio of the gyromagnetic ratios of the free electron and proton γ*_S_*/γ*_I_*, with a maximum value of 660 [21].

### 2.3. Larmor Precession

**Figure 3:**
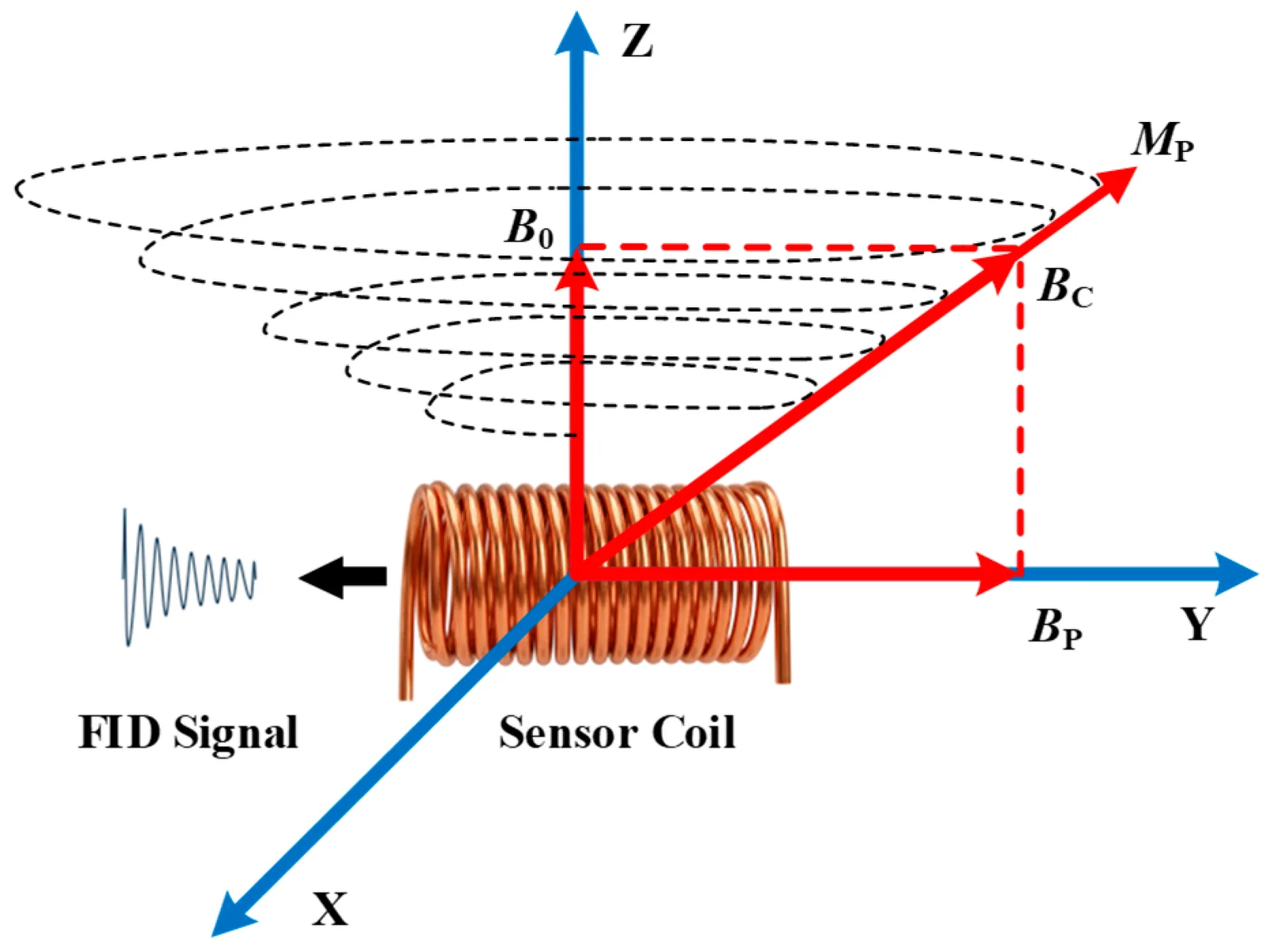
Larmor precession principle.

By applying a polarization current to the sensor coil, a bias polarization magnetic field ***B***_p_ is generated. Under the combined effect of the polarization field ***B***_p_ and the geomagnetic field ***B***_0_, the macroscopic magnetic moment of the hydrogen protons in the free-radical solution will align along the resultant vector, producing a resultant magnetic moment ***M***_P_. After polarizing for a period of time with the radio-frequency signal and polarization current, the radio-frequency and polarization current polarization signals are turned off. The resultant magnetic moment ***M*_P_** of the protons precess around the geomagnetic field ***B***_0_. During the precession process, relaxation occurs. The protons cut through the magnetic field lines of the coil, generating an electric signal, which is the FID (Free Induction Decay), as shown in Figure 3. The frequency *f*_0_ of this signal satisfies the following equation in relation to the Earth’s field [22,23]:

2*πf*_0_ = *γ*_P_*B*_0_ (3)

where *γ*_P_ is the gyromagnetic ratio of the hydrogen proton nucleus, with a value of 2.67515255 × 10^8^ rad s^−1^ T^−1^. By measuring the frequency of the Larmor precession signal, the magnitude of the geomagnetic field can be determined.

## 3. Construction of Marine Base Station OVM

### 3.1. Brief Introduction of the Marine Base Station OVM

**Figure 4:**
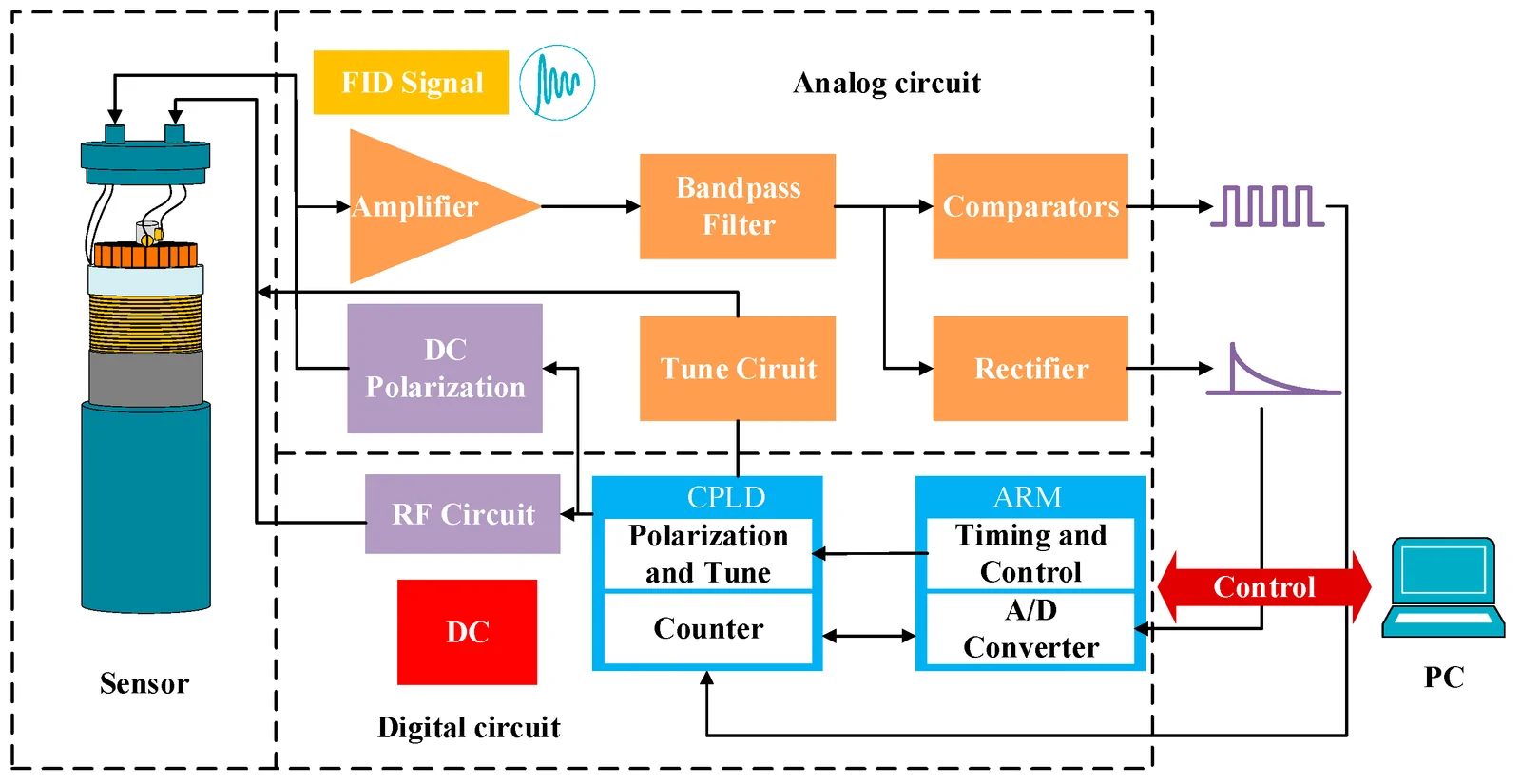
Block diagram of the marine base station OVM.

**Figure 5:**
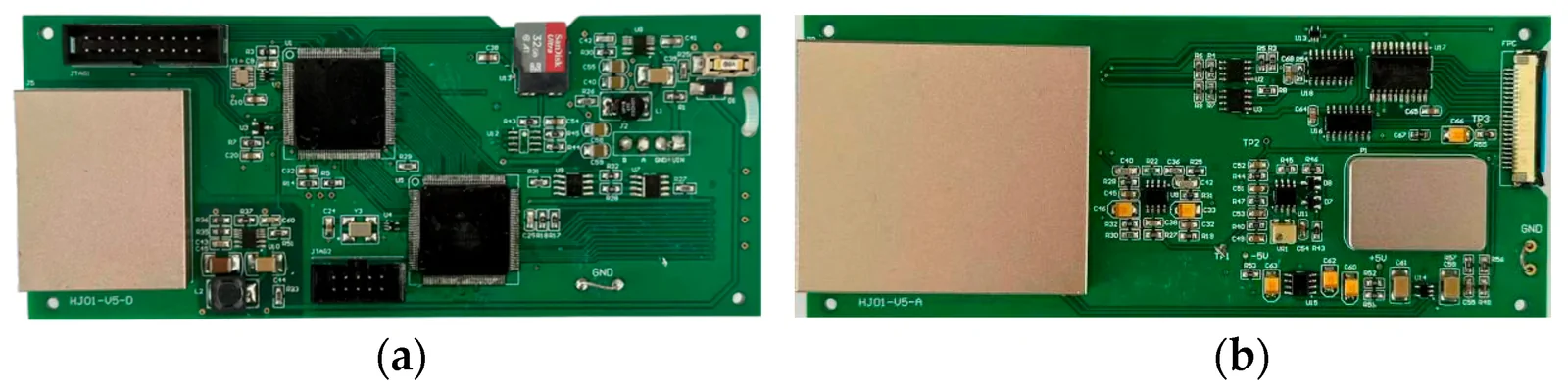
(**a**) Digital board. (**b**) Analog board.

The schematic block diagram of the marine base station OVM is shown in Figure 4. The system consists of a sensor and an electronics module. The electronics module, including the analog circuit and digital circuit shown in Figure 5, is enclosed in shielded boxes to prevent electromagnetic interference. The analog circuit includes modules such as the polarization tuning circuit, amplifier circuits, envelope detection circuit, and shaping circuit. The digital circuit comprises the power circuit, CPLD, ARM, and RF circuits.

The system communicates with a host computer via the RS-485 interface for parameter setup and data transmission. After setting the cycle time, tuning mode, and RTC (Real-Time Clock) time synchronization, the system is fully configured. When the magnetometer starts to acquire a reading, the ARM processor controls the CPLD to execute RF polarization, DC polarization, and tuning control. The sensor receives the FID (Free Induction Decay) signal after polarization. Then, the tuning capacitor in the tuning circuit, along with the sensor coil’s inductance, forms an LC series resonance circuit, which filters the FID signal. The amplified FID signal is processed by a comparator to generate a digital frequency signal, which is then sent to the CPLD to count the frequency of the FID signal. The rectified signal is transmitted to the ARM’s A/D converter to evaluate the FID signal’s quality.

**Figure 6:**
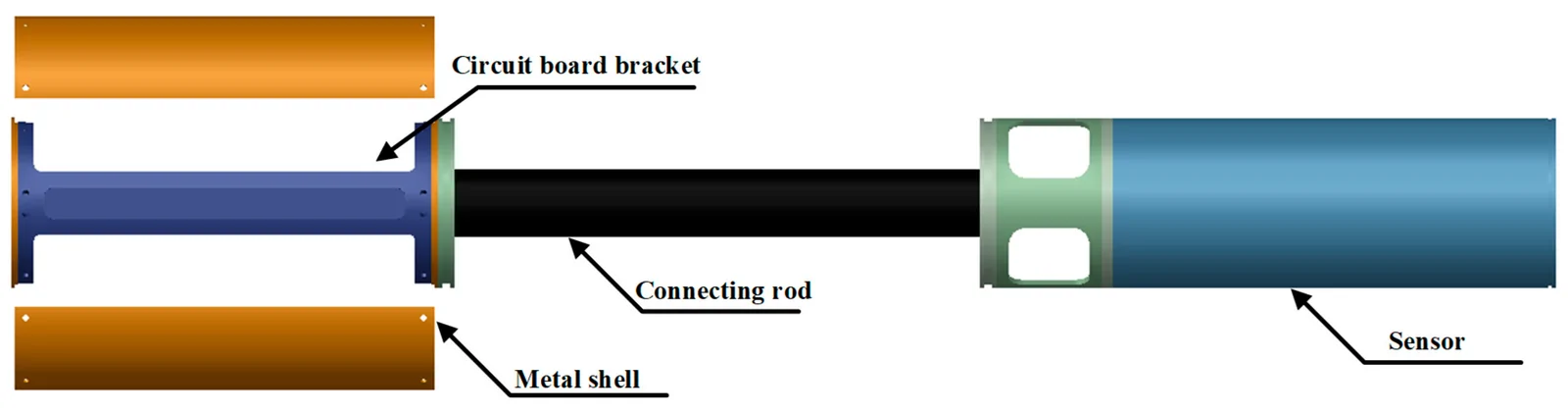
Structural block diagram.

The internal compartment of the magnetometer includes the sensor and the circuit board bracket, which are connected via a rod. To ensure electromagnetic shielding, the circuit board bracket is enclosed within a metal shell. The layout of the internal compartment is shown in Figure 6.

Furthermore, the overall average power consumption of the developed magnetometer during continuous operation is tightly controlled at approximately 38 mW for a 60 s cycle time, effectively meeting the ultra-low power requirements for long-term uninterrupted seabed observation.

### 3.2. Sensor Design

**Figure 7:**
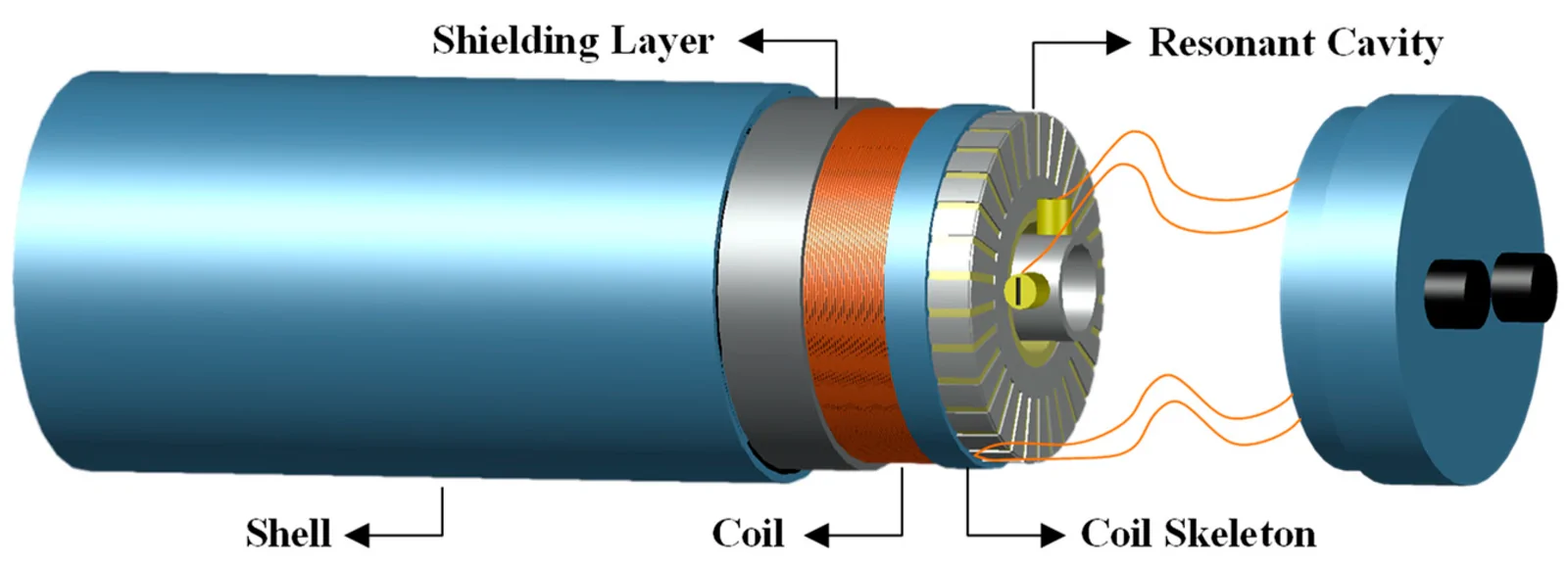
Sensor structure diagram.

The core of the OVM is the sensor, which consists of two parts: an RF coaxial resonant cavity and an omnidirectional coil, with an electronic and nuclear double resonance working material. The coaxial resonant cavity is used to generate a uniform RF polarization magnetic field, causing the unpaired electrons in the free-radical solution to undergo electron spin resonance. The omnidirectional coil is wrapped around the outside of the resonant cavity, generating a DC polarization magnetic field and receiving the FID signal. The structural diagram is shown in Figure 7.

#### 3.2.1. Coaxial Resonant Cavity

**Figure 8:**
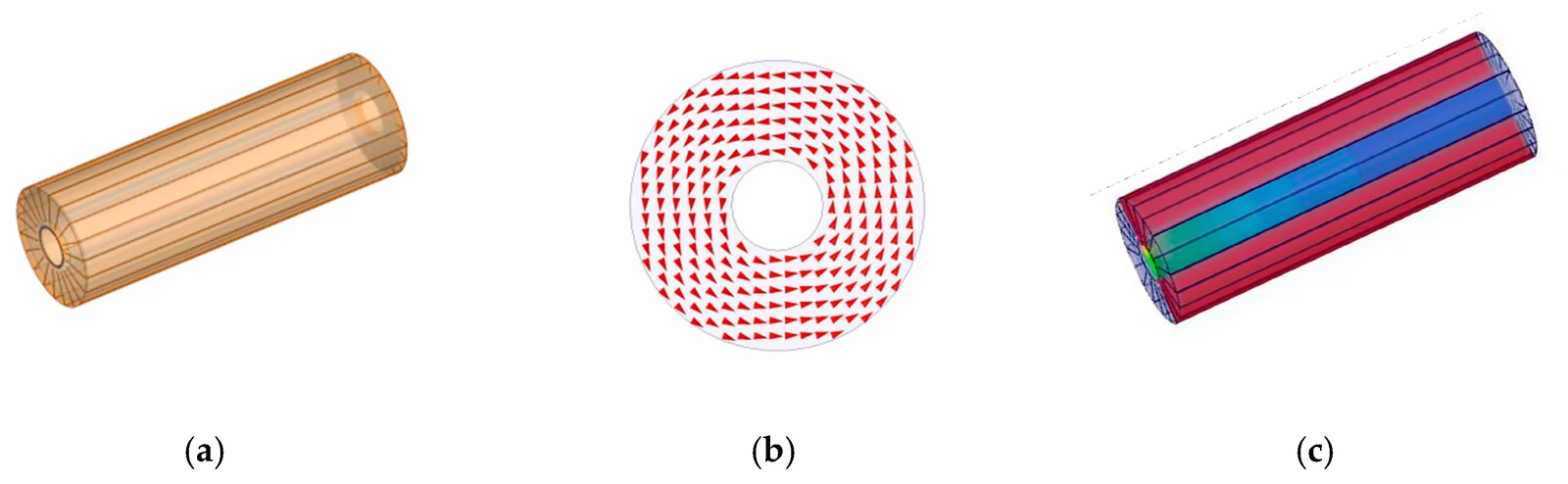
(**a**) HFSS model of resonant cavity. (**b**) Cross-sectional magnetic field distribution. (**c**) Longitudinal magnetic field distribution.

The coaxial resonant cavity consists of an ampoule flask filled with a free-radical solution and metal foil strips connected to the inside and outside of the container. When an RF signal is applied to the resonant cavity, a uniform circularly polarized magnetic field is formed in the free-radical solution region, as shown in Figure 8b,c. To avoid eddy-current induction in the resonant cavity’s outer conductor caused by the magnetic field from the DC polarizing current, the outer conductor is fabricated using strips of copper foil separated by 2 mm gaps.

**Figure 9:**
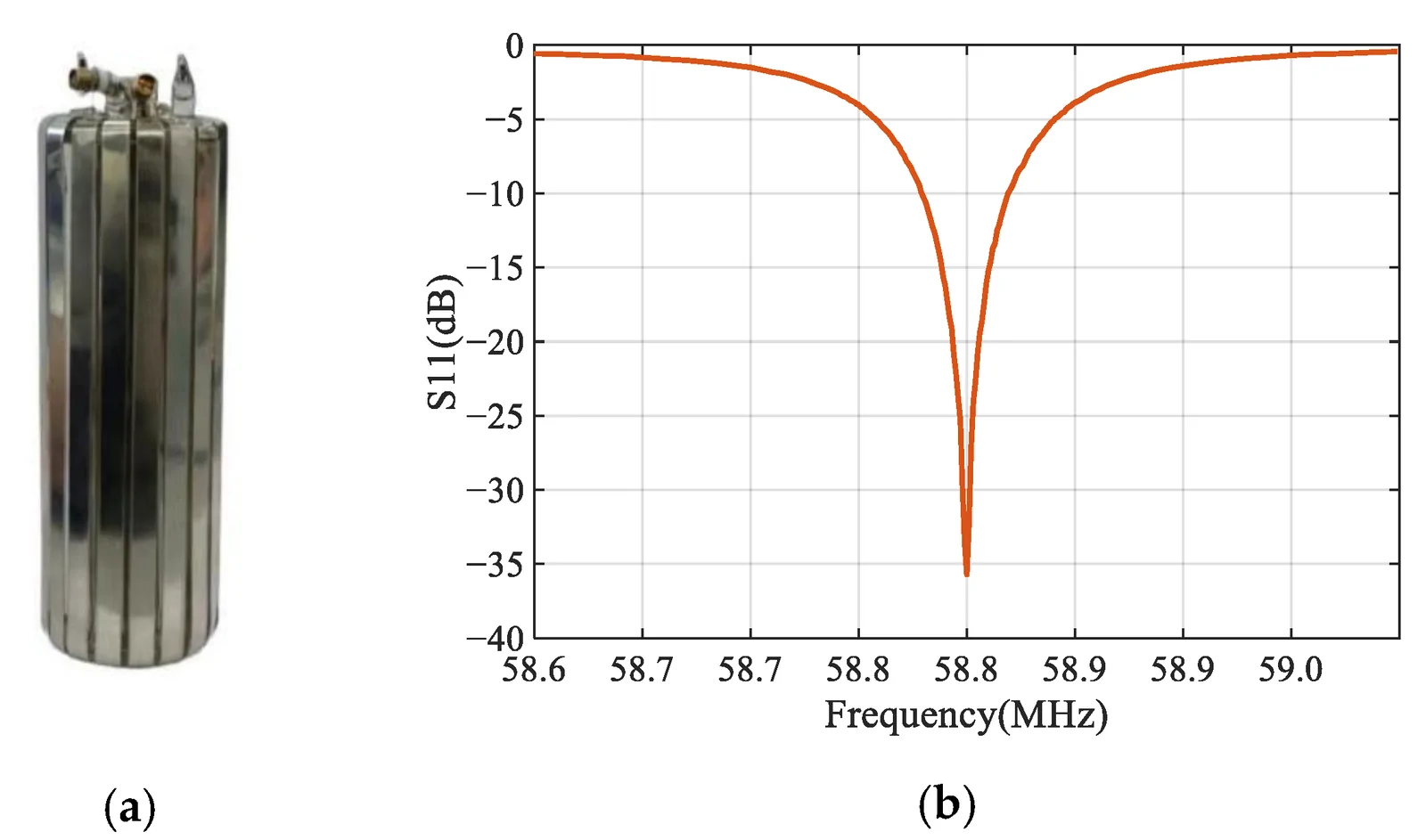
(**a**) Physical diagram of the resonant cavity. (**b**) S11 parameters of the resonant cavity.

As shown in Figure 9a, two variable capacitors are used on the top of the sensor, one in series and the other in parallel to the sensor. The parallel capacitors are responsible for adjusting the resonant frequency, while the series capacitors are responsible for adjusting the S parameter. After carefully adjusting the capacitors, the measured S11 parameter, as shown in Figure 9b, is approximately −36 dB, with a center frequency of 58.8 MHz. The equation for calculating the Q-factor is

(4) $$Q=\frac{f_{0}}{\Delta f}=\frac{f_{0}}{f_{h}-f_{l}}$$

where *f*_0_ = 58.8 MHz is the resonant frequency and *f*_l_ and *f*_h_ are lower and upper cutoff frequencies at −10 dB, respectively. The measured *f*_l_ and *f*_h_ are 58.782 MHz and 58.823 MHz, respectively. The calculated Q value is 1434.

#### 3.2.2. Omnidirectional Coil

**Figure 10:**
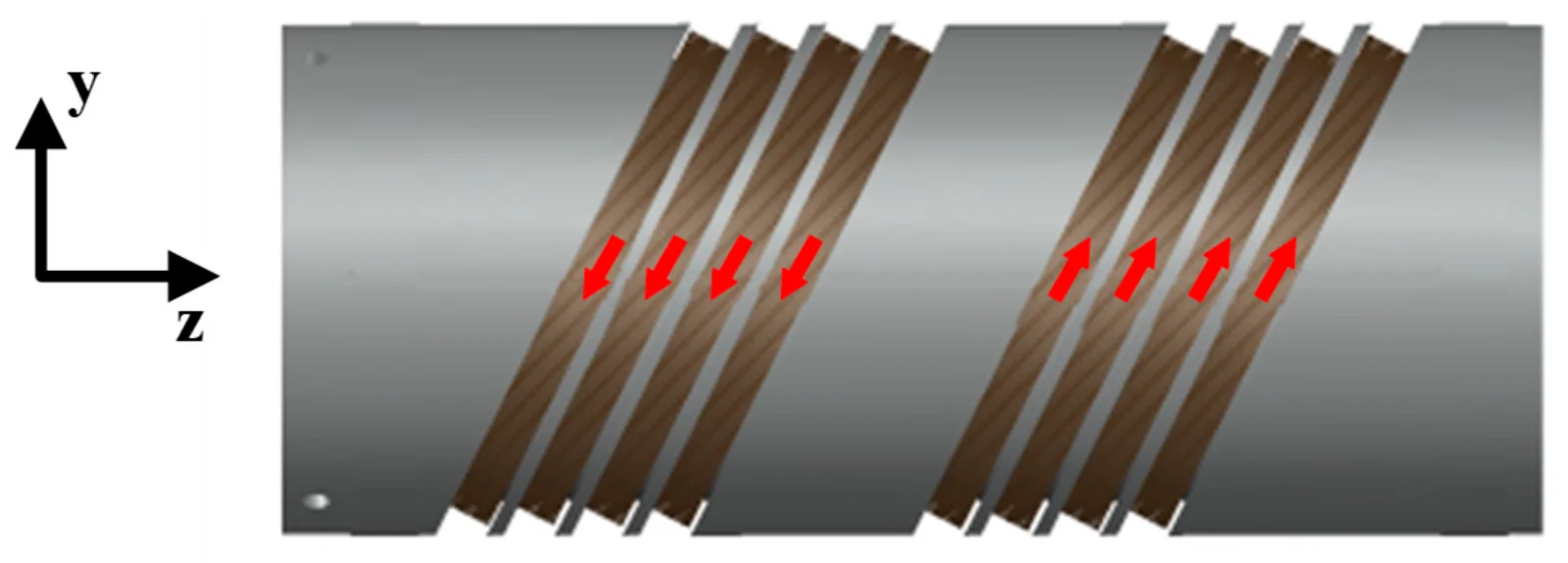
Schematic diagram of the eight-section coil.

Unlike portable magnetometers, where the sensor’s orientation can be easily adjusted, marine magnetometers are usually deployed underwater, and it is difficult to adjust the sensor’s orientation. When the angle *θ* between the Earth’s field and the polarized nuclear magnetization decreases, the signal amplitude diminishes. The quantitative relationship between the Larmor signal and *θ* can be expressed by the following equation [24]:

(5) V(t)=μ0nAMω0e−tTsinω0t sin2θ

where μ_0_ is the vacuum magnetic permeability, *A* is the area of the coil, *n* is the number of turns in the coil, *M* is the nuclear magnetization strength, *T* is the relaxation time, *ω*_0_ is the Larmor precession frequency, and *θ* is the angle between the magnetic field to be measured and *M*. From Equation (5), it can be seen that the amplitude of the Larmor signal depends on *θ*. When *M* is parallel to the direction of the magnetic field being measured, *θ* = 0°, and the Larmor signal theoretically approaches zero. This direction is referred to as the measurement dead zone. To avoid such extreme situations, an inclined coil structure is proposed to produce both mutually orthogonal axial and radial magnetic field components. In order to increase the inclination and reduce the diameter of the coil, an eight-segment coil is used to enhance the radial component, as shown in Figure 10.

As shown in Figure 10, the low-frequency coil system consists of two sets of coils wound in opposite directions. This design reduces external electromagnetic interference by generating opposing induced EMFs and improves the coil’s resistance to interference. During the reception process, after the hydrogen protons in the free radicals are polarized, their magnetic moments are opposite in the two sets of coils. As these moments precess around the Earth’s magnetic field, the phase difference of the induced EMF is 180°. Because the winding directions of the two sets of coils are opposite, the EMFs of the Larmor signal generated by the coils will overlap and increase the amplitude of the signal.

To design an omnidirectional sensor, it is necessary to quantitatively study the relationship between the components of the polarized magnetic field in two mutually orthogonal directions. Therefore, we defined a new parameter, the omnidirectional coefficient *O*_m_, which can be written as follows:

(6) $$O_{m}=\min\left\{\frac{\sum_{i=1}^{n}\left|B_{vi}\right|}{\sum_{i=1}^{n}\left|B_{ai}\right|},\frac{\sum_{i=1}^{n}\left|B_{ai}\right|}{\sum_{i=1}^{n}\left|B_{vi}\right|}\right\}$$

where ***B*_vi_** and ***B*_ai_** denote the radial and axial magnetic field components at each point within the solution region, respectively. The omnidirectionality coefficient reflects the orthogonality of the polarizing magnetic field. A polarizing magnetic field with a single-directional orientation yields the minimum omnidirectionality coefficient of 0, which corresponds to the so-called dead zone. Conversely, when the two mutually orthogonal components of the polarizing magnetic field are equal, the omnidirectionality coefficient reaches its maximum value of 1. A detailed calculation of the omnidirectional coefficient is presented below.

**Figure 11:**
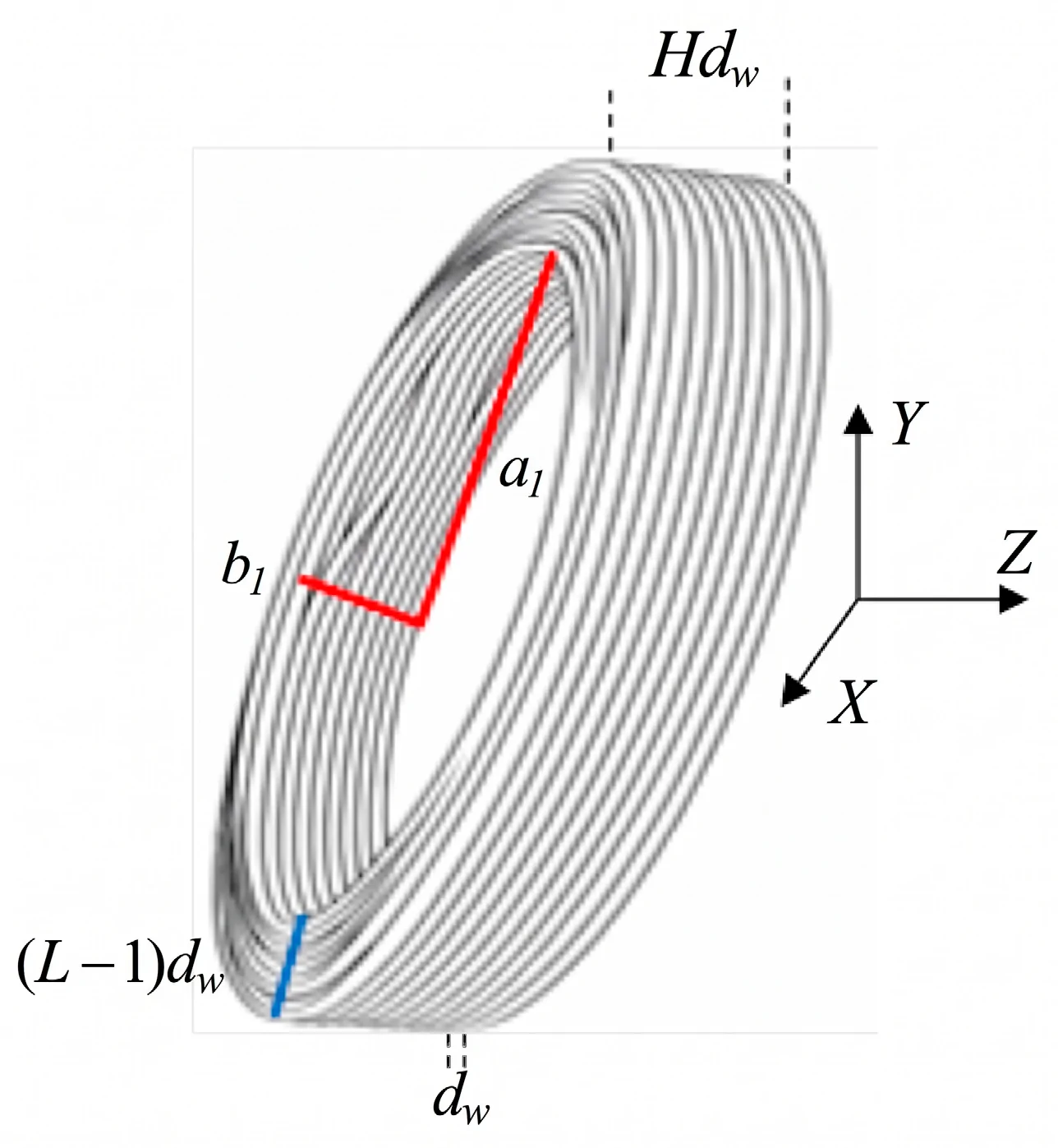
Calculation of elliptic inclined coil group.

One of the eight inclined coils is shown in Figure 11, and each individual coil is composed of multiple layers of radially nested elliptical rings. For the *l*-th (*l* = 1, 2, …, *L*) coil layer, the expressions for its elliptical major axis *a_l_* and minor axis *b_l_* are given by

(7) $$\left\{\begin{array}{l} a_{l}=\frac{R}{\mathrm{cos\theta}}+(l-1)d_{w}\\ b_{l}=R+(l-1)d_{w} \end{array}\right.$$

where *R* is the baseline inner radius of the solenoid, *d*_w_ represents the wire diameter, and *θ* denotes the inclined angle of the entire coil assembly around the X-axis.

Each layer of the circular coil is uniformly discretized along the circumferential direction into *N* current element sampling points. The discrete angle of the *j*-th sampling point is given by

(8) $$\varphi_{j}=\frac{2\pi (j-1)}{N}(j=1,2,\ldots ,N)$$

In the XY plane, the position vector ***r****_l_*_,*j*_ and the normalized unit tangent vector *d**l**_l_*_,*j*_ of this current element are expressed as follows, respectively:

***r**_l_*_,*j*_ = (*b_l_*cos*φ_j_*,*a_l_*sin*φ_j_*,0) (9)

(10) $$dl_{l,j}=\left(-\frac{b_{l}\sin\varphi_{j}}{\left|-b_{l}\sin\varphi_{j},a_{l}\cos\varphi_{j}\right|},\frac{a_{l}\cos\varphi_{j}}{\left|-b_{l}\sin\varphi_{j},a_{l}\cos\varphi_{j}\right|},0\right)$$

Considering that the coil group rotates around the X-axis by *θ* and is axially stacked by *H* identical coil groups along the Z-axis with a spacing equal to the wire diameter *d*_w_, the final geometric position coordinates ***r_g,l,j_*** and the final tangent direction vector *d**l_g,l,j_*** in the three-dimensional space for the *j*-th current element of the *l*-th layer in the *g*-th group (*g* = 1,2, …, *H*) are respectively expressed as follows:

***r_g_*_,*l*,*j*_** = (*b_l_cosφ_j_*,*a_l_sinφ_j_*cos*θ*,*a_l_*sin*φ_j_*sin*θ* + (*g* − 1)*d*_w_) (11)

(12) $$dl_{g,l,j}=\left(-\frac{b_{l}\sin\varphi_{j}}{\left|-b_{l}\sin\varphi_{j},a_{l}\cos\varphi_{j}\right|},\frac{a_{l}\cos\varphi_{j}\cos\theta }{\left|-b_{l}\sin\varphi_{j},a_{l}\cos\varphi_{j}\right|},\frac{a_{l}\cos\varphi_{j}\sin\theta }{\left|-b_{l}\sin\varphi_{j},a_{l}\cos\varphi_{j}\right|}\right)$$

According to the Biot–Savart law, the total magnetic field ***B***(***r_p_***) at any arbitrary spatial observation target point ***r****_p_*(*x_p_*,*y_p_*,*z_p_*) can be obtained by superimposing the contributions from all discrete current elements:

(13) $$B(r_{p})=\sum_{g=1}^{H}\sum_{l=1}^{L}\sum_{j=1}^{N}\frac{\mu_{0}}{4\pi }\frac{\mathrm{Id}l_{g,l,j}\times (r_{p}-r_{g,l,j})}{{\left|r_{p}-r_{g,l,j}\right|}^{3}}$$

where μ_0_ is the vacuum magnetic permeability and *I* is the excitation current applied to a single-turn coil. To evaluate the magnetic field distribution characteristics on a specific observation surface, this study focuses on the YZ observation plane (i.e., *x_p_* = 0). By substituting Equations (11) and (12) into Equation (13) and expanding the vector cross product, the fully expanded analytical expressions for the independent magnetic field components ***B****_y_* and ***B_z_*** at any arbitrary observation point on the YZ plane can be derived, respectively:

(14) $$B_{y}(0,y_{p},z_{p})=\sum_{g=1}^{H}\sum_{l=1}^{L}\sum_{j=1}^{N}\frac{\mu_{0}I}{4\pi }\frac{b_{l}\left[z_{p}\sin\varphi_{j}-a_{l}\sin\theta -(g-1)d_{w}\sin\varphi_{j}\right]}{\sqrt{b_{l}^{2}\mathrm{si}n^{2}\varphi_{j}+a_{l}^{2}\mathrm{co}s^{2}\varphi_{j}}\cdot D_{g,l,j}^{3/2}}$$

(15) $$B_{z}(0,y_{p},z_{p})=\sum_{g=1}^{H}\sum_{l=1}^{L}\sum_{j=1}^{N}\frac{\mu_{0}I}{4\pi }\frac{b_{l}\left(a_{l}\cos\theta -y_{p}\sin\varphi_{j}\right)}{\sqrt{b_{l}^{2}\mathrm{si}n^{2}\varphi_{j}+a_{l}^{2}\mathrm{co}s^{2}\varphi_{j}}\cdot D_{g,l,j}^{3/2}}$$

where *D_g_*_,*l*,*j*_ is the distance variable and its value is given by

*D_g_*_,*l*,*j*_=(*b_l_*cos*φ_j_*)^2^+(*y_p_* − *y_g_*_,*l*,*j*_)^2^+(*z_p_* − *z_g_*_,*l*,*j*_)^2^ (16)

In *D_g_*_,*l*,*j*_, the formula corresponding to the position of the current element is given by

(17) $$\left\{\begin{array}{l} y_{g,l,j}=a_{l}\sin\varphi_{j}\cos\theta \\ z_{g,l,j}=a_{l}\sin\varphi_{j}\sin\theta +(g-1)d_{w} \end{array}\right.$$

The solution region occupies a specific volume, which can be sliced into *K* reference planes to separately calculate the ***B****_y_* and ***B**_z_* components for each plane. Furthermore, the continuous angle *η* can be discretized into a specific sequence of observation orientations:

(18) $$\eta_{k}=\frac{2\pi (k-1)}{K}(k=1,2,\ldots ,K)$$

where *K* is the total number of discrete reference planes. For the *k*-th discrete orientation reference plane (with a rotation angle of *η_k_*), the orthogonal magnetic field components at any arbitrary point on the Y′Z′ observation plane are calculated by the following formulas:

(19) $$B_{y^{’}}(0,y_{p}’,z_{p}’,\eta_{k})=\sum_{g=1}^{H}\sum_{l=1}^{L}\sum_{j=1}^{N}\frac{\mu_{0}I}{4\pi }\frac{\lambda_{y’,k}(g,l,j)}{{\left[D_{k}(g,l,j)\right]}^{3/2}}$$

(20) $$B_{z^{’}}(0,y_{p}’,z_{p}’,\eta_{k})=\sum_{g=1}^{H}\sum_{l=1}^{L}\sum_{j=1}^{N}\frac{\mu_{0}I}{4\pi }\frac{b_{l}\left[a_{l}\mathrm{cos\theta}-(y_{p}’\cos\eta_{k})\sin\varphi_{j}\right]}{\sqrt{b_{l}^{2}\mathrm{si}n^{2}\varphi_{j}+a_{l}^{2}\mathrm{co}s^{2}\varphi_{j}}\cdot {\left[D_{k}(g,l,j)\right]}^{3/2}}$$

in which the vector projection function *λ_y’_*_,*k*_(*g*,*l*,*j*) of the *k*-th reference plane is defined as

(21) $$\lambda_{y’,k}(g,l,j)=\frac{\sigma_{j,k}\sin\eta_{k}+\varsigma_{j,k}\cos\eta_{k}}{\sqrt{b_{l}^{2}\sin^{2}\varphi_{j}+a_{l}^{2}\mathrm{co}s^{2}\varphi_{j}}}$$

where the algebraic coupling coefficients *σ_j_*_,*k*_ and ς*_j_*_,*k*_ corresponding to each reference plane are respectively expressed as

(22) $$\left\{\begin{array}{l} \sigma_{j,k}=(a_{l}\cos\varphi_{j}\cos\theta )\cdot \left[z_{p}’-a_{l}\sin\varphi_{j}\sin\theta -(g-1)d_{w}\right]\\ -(a_{l}\cos\varphi_{j}\sin\theta )\cdot \left[y_{p}’\cos\eta_{k}-a_{l}\sin\varphi_{j}\cos\theta \right]\\ \varsigma_{j,k}=(a_{l}\cos\varphi_{j}\sin\theta )\cdot \left[-y_{p}’\sin\eta_{k}-b_{l}\cos\varphi_{j}\right]\\ +(b_{l}\sin\varphi_{j})\cdot \left[z_{p}’-a_{l}\sin\varphi_{j}\sin\theta -(g-1)d_{w}\right] \end{array}\right.$$

In this study, the radial direction of the coil corresponds to the ***B**_y_*′ direction, while the axial direction corresponds to the ***B**_z_*′ direction. According to Equations (6), (19) and (20), the omnidirectionality coefficient formula for the inclined coil group can be expressed as

(23) $$O_{m}=\frac{\sum_{i=1}^{n}\left|B_{y’i}\right|}{\sum_{i=1}^{n}\left|B_{z’i}\right|}$$

where ***B**_y_*′ and ***B**_z_*′ are the Y-component and Z-component of the magnetic field generated by the coil group at each point within the solution region, respectively.

**Figure 12:**
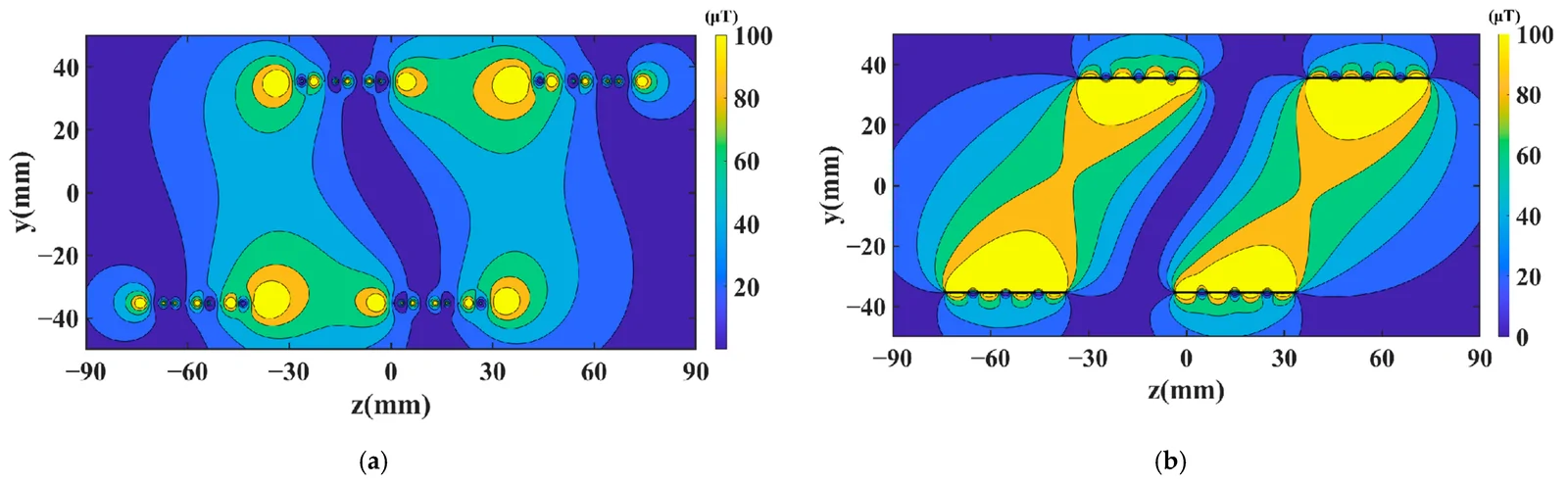
(**a**) The radial magnetic fields generated by the inclined coil. (**b**) The axial magnetic fields generated by the inclined coil.

According to Equations (14) and (15), by summing the contributions from each layer of the coil along the Z-axis, the overall magnetic field distribution produced by the coil at any point on the YZ plane can be obtained (with the Y-axis perpendicular to the axial direction and the Z-axis parallel to the axial direction). The calculated results for a random rotation angle are illustrated in Figure 12.

**Figure 13:**
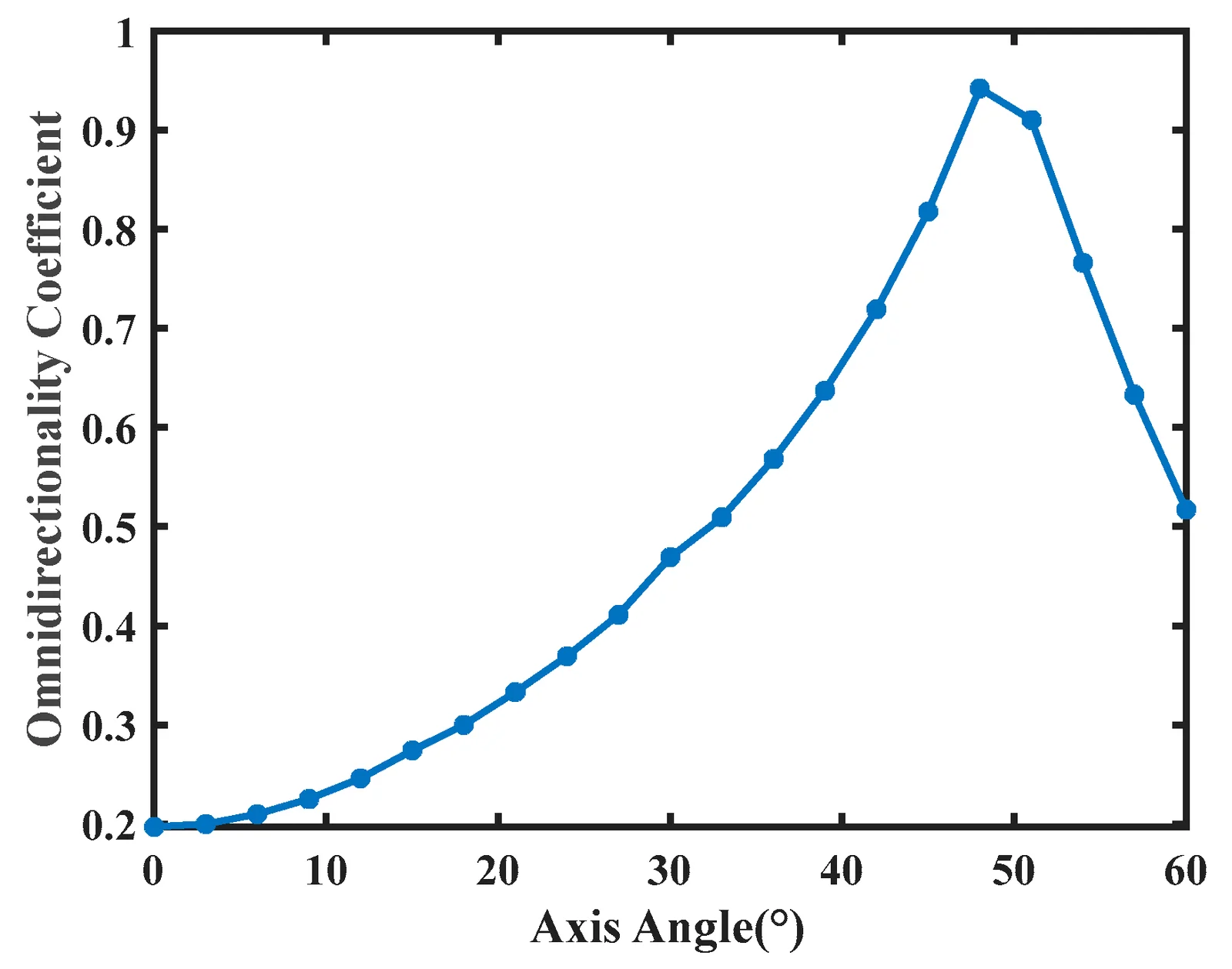
Omnidirectionality coefficient vs. inclined angles of the inclined coil.

From Figure 12, we can see that the expected radial polarized magnetic fields are obtained due to the inclined coils, and the increased radial magnetic field will help improve the omnidirectional performance of the sensor. To evaluate the performance, various inclination angles *θ* ranging from 0° to 60° were investigated to calculate the corresponding omnidirectionality coefficient of the low-frequency coil within the solution region. According to Equation (23), the calculated results are summarized in Figure 13.

Figure 13 illustrates the variation trend of the omnidirectionality coefficient as a function of the coil inclined angle from 0° to 60°. As the inclined angle increases beyond 15°, the curve exhibits an accelerated upward trend. The maximum value of the omnidirectional coefficient occurs at approximately 48° and then sharply decreases.

**Figure 14:**
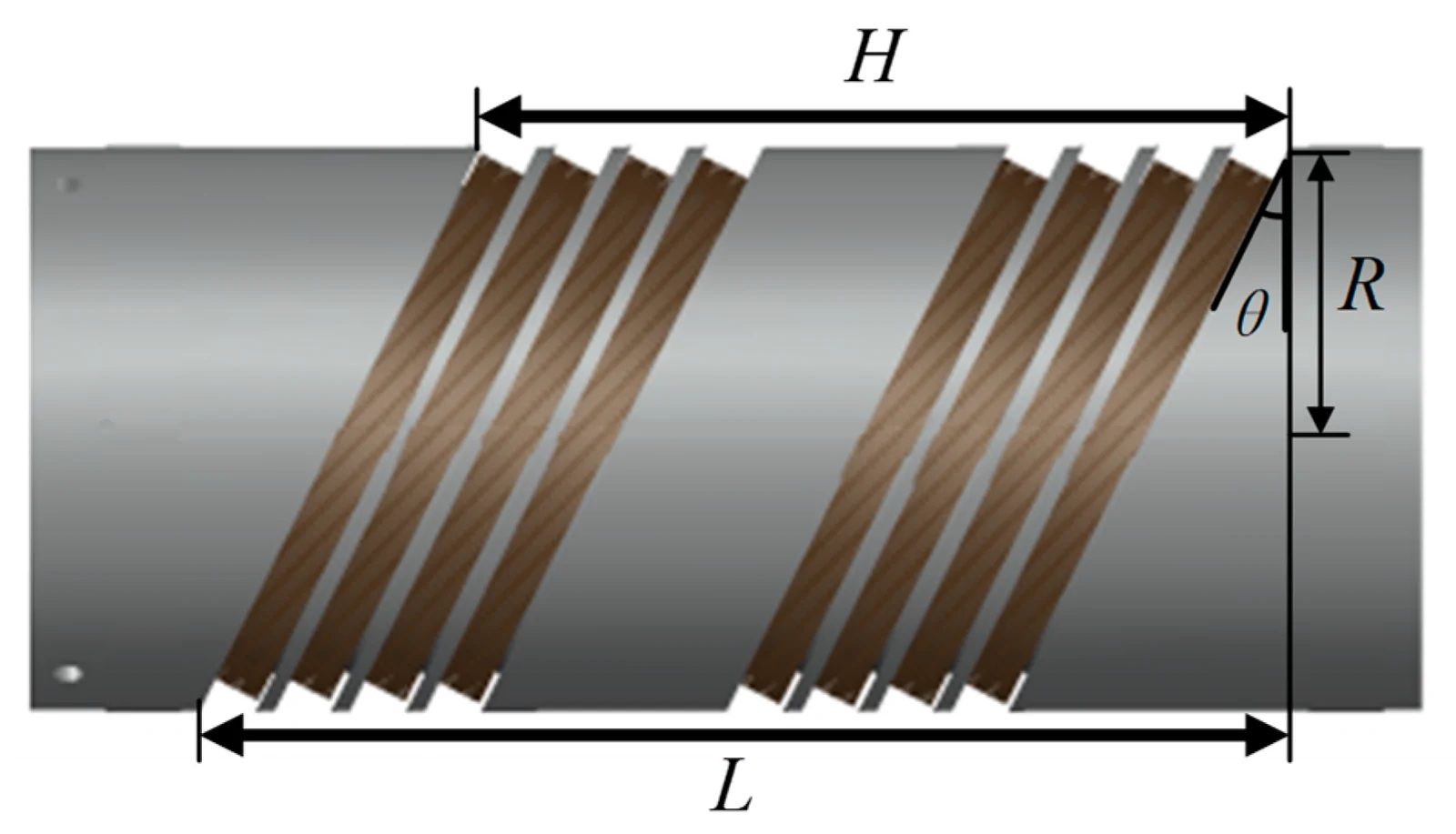
Dimensions of the inclined coil.

The quantitative calculation formula is presented below. Assuming a total coil height of *H* and a fixed radial dimension of *R*, as illustrated in Figure 14, the relationship between the axial dimension *L* and the inclined angle *θ* is expressed as

*L*(*θ*) = *H* + 2*R*tan*θ* (24)

**Figure 15:**
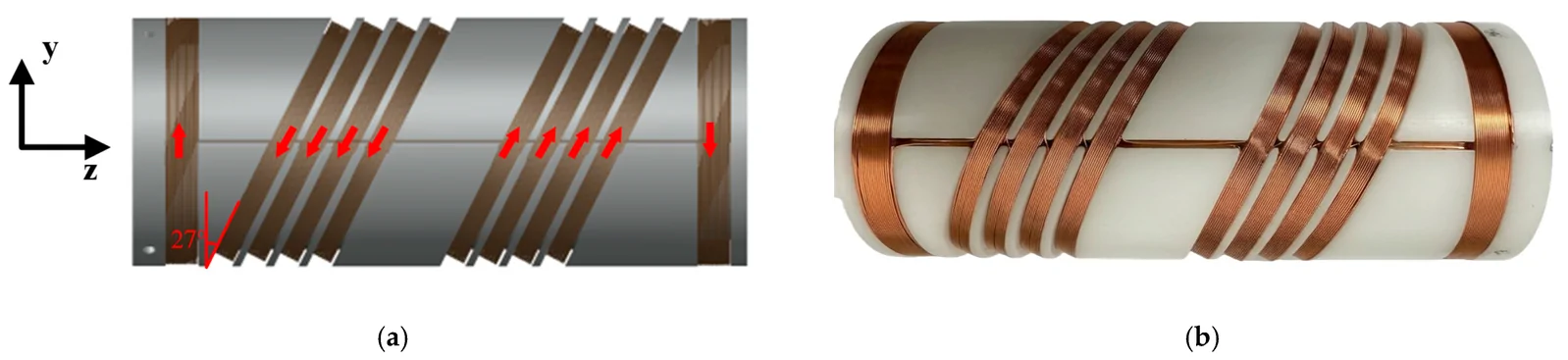
(**a**) Model of the omnidirectional coil along with compensation coils on both sides. (**b**) Image of the omnidirectional coil.

Considering that a 48° inclined angle results in an excessively large sensor volume, the 27° inclined angle is used in our design. However, the inclined angle of the inclined coil is too small to provide sufficient radial polarization magnetic field components, which limits the improvement of the omnidirectional coefficient. In order to further improve the omnidirectionality, two reversed compensation coils with axial winding are added at both ends of the inclined coil. The schematic diagram and physical diagram are shown in Figure 15.

The calculation method for the polarizing magnetic field generated by the compensation coil is identical to that for the tilted coil. For a compensation coil with an initial radius *R* and a total of *L*′ radial layers, the *l*′-th ring (*l’* = 1, 2, …, *L*′) can be expressed as

*C_l_*_’_ = *R* + (*l*’ − 1)*d_w_* (25)

Based on Equations (8) and (9), each layer of the compensation coil is uniformly discretized along the circumference into *N* current-element sampling points. The coordinates of the corresponding current element in the XY plane, **r***_l’_*_,*j*_, and the unit tangential vector along the current element d**l***_l’_*_,*j*_ are given by

***r**_l_*_’,*j*_ = (*C_l_*_’_cos*φ_j_*, *C_l_*_’_sin*φ_j_*, 0) (26)

*d**l**_l_*_’,*j*_ = (−sin*φ_j_*, cos*φ_j_*, 0) (27)

Similarly, when the compensation coil is stacked with *H*′ layers along the Z-axis, the coordinates of the g*’*-th layer (*g*′ = 1,2, …, *H*′) in the *Z*-direction are expressed as

(28) $$r_{g^{’},l^{’},j}=\left(C_{l’}\cos\varphi_{j},C_{l’}\sin\varphi_{j},(g’-1)d_{w}\right)$$

According to Equation (13), the radial and axial magnetic field components of the compensation coil can be derived as follows:

(29) $$B_{\mathrm{cy}}(0,y_{p},z_{p})=\sum_{g’=1}^{H’}\sum_{l’=1}^{L’}\sum_{j=1}^{N}\frac{\mu_{0}I}{4\pi }\frac{C_{l’}\sin\theta_{j}z_{p}}{{\left(D_{g^{’},l^{’},j}\right)}^{3/2}}$$

(30) $$B_{\mathrm{cz}}(0,y_{p},z_{p})=\sum_{g’=1}^{H’}\sum_{l’=1}^{L’}\sum_{j=1}^{N}\frac{\mu_{0}I}{4\pi }\frac{C_{l’}^{2}-C_{l’}y_{p}\sin\theta_{j}}{{\left(D_{g^{’},l^{’},j}\right)}^{3/2}}$$

where the squared distance denominators are respectively defined as

(31) $$D_{g^{’},l^{’},j}=C_{l^{’}}^{2}+y_{p}^{2}-2C_{l’}y_{p}\sin\theta_{j}+z_{p}^{2}$$

According to the principle of field superposition, the radial and axial magnetic field components of the total coil system after incorporating the compensation coils are expressed as

(32) $$\left\{\begin{array}{l} B_{v}=B_{y^{’}}+B_{\mathrm{cy}}\\ B_{a}=B_{z}^{’}+B_{\mathrm{cz}} \end{array}\right.$$

**Figure 16:**
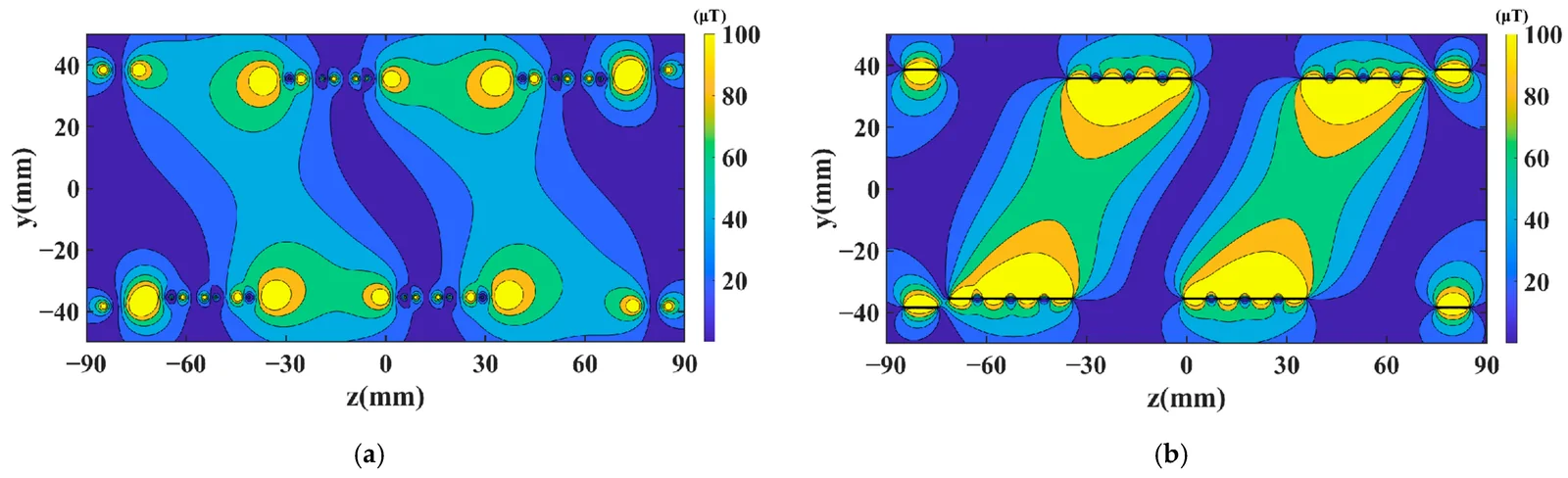
(**a**) The radial magnetic fields generated by the inclined coil along with compensation coils on both sides. (**b**) The axial magnetic fields generated by the inclined coil along with compensation coils on both sides.

Based on Equation (32), the radial and axial magnetic field components of the introduced compensation coils are calculated and illustrated in Figure 16.

As shown in Figure 16, the introduction of the compensation coils induces a radial magnetic field component within the gap between these coils and the inclined coils while simultaneously weakening the axial magnetic field component at the centerline of the Y-axis. Therefore, adding compensation coils aims to balance the orthogonal field strengths to significantly improve the sensor’s omnidirectionality coefficient.

**Figure 17:**
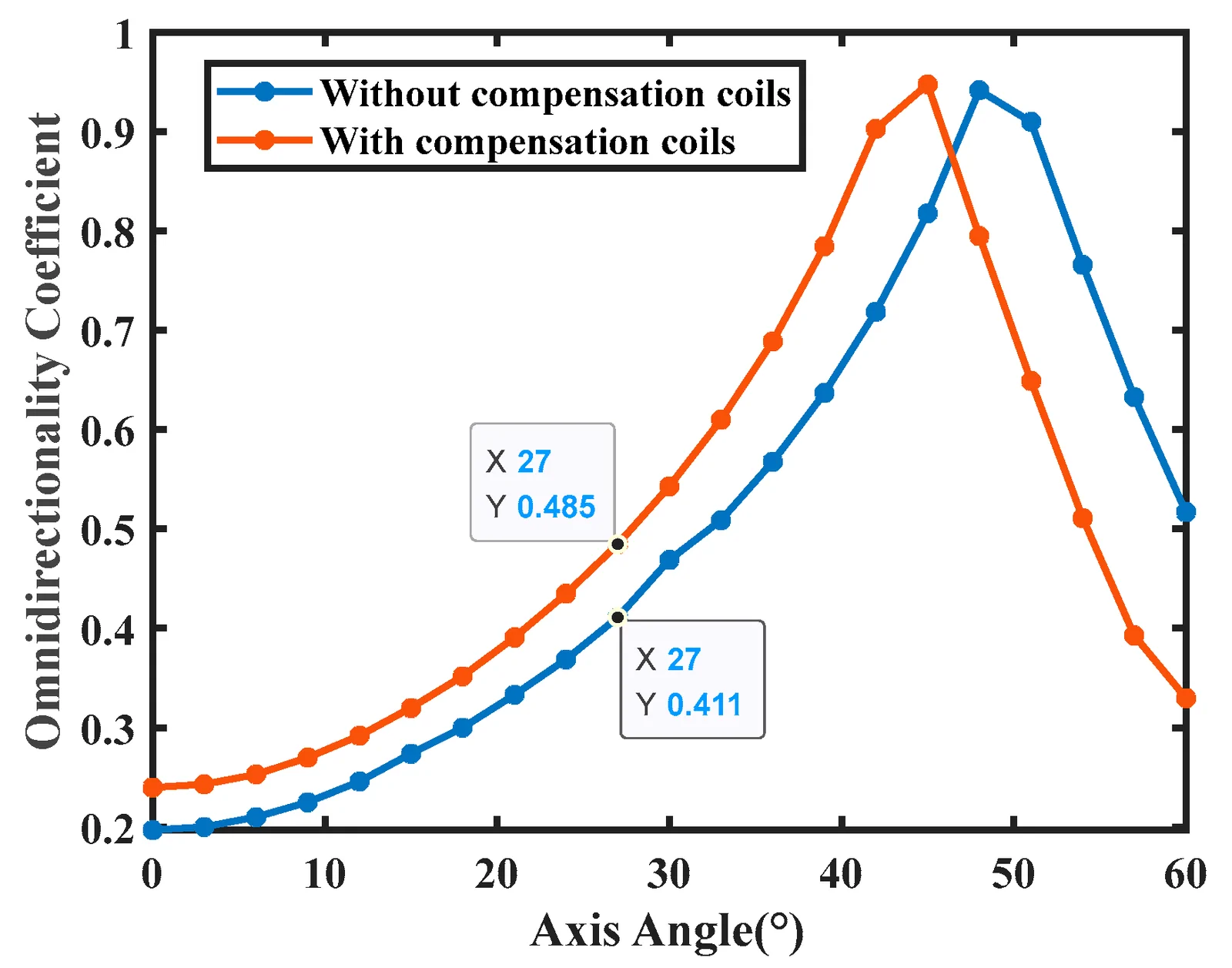
Omnidirectionality coefficient of different inclined angles of an omnidirectional coil with compensation coils and without compensation coils.

To evaluate the performance of the compensation coils, the omnidirectionality coefficient for each coil inclined angle consisting of the compensation coils was obtained. As shown in Figure 17, with the addition of the compensation coils, the omnidirectionality coefficient of the inclined coil increased, and at an angle of 27°, the omnidirectionality coefficient increases from 0.411 to 0.485.

## 4. Performance Test and Experimental Verification

### 4.1. The Omnidirectionality Test

As described above, we have defined the omnidirectional coefficient and proposed an omnidirectional sensor with a compensation coil structure. Below, we will measure the omnidirectional coefficient of the sensor and demonstrate its omnidirectional performance.

Under conventional conditions, the magnetization is linearly proportional to the polarization magnetic field strength [23]. According to Equation (6), the relationship between the omnidirectionality coefficient and the components of the polarization magnetic field can be established. Consequently, the relationship between the omnidirectionality coefficient and the components of the magnetization is derived. Furthermore, combining this with the relationship between the Larmor signal amplitude and the magnetization given in Equation (5), the quantitative connection between the omnidirectionality coefficient and the initial amplitude of the Larmor signal is ultimately obtained:

(33) $$O_{m}=\frac{\sum_{i=1}^{n}\left|B_{\mathrm{vi}}\right|}{\sum_{i=1}^{n}\left|B_{\mathrm{ai}}\right|}=\frac{\sum_{i=1}^{n}\left|M_{\mathrm{vi}}\right|}{\sum_{i=1}^{n}\left|M_{\mathrm{ai}}\right|}=\frac{V_{\min}}{V_{\max}}$$

where ***M***_vi_ and ***M***_ai_ denote the radial and axial magnetization components at each point within the solution region, respectively, and *V*_min_ (or *V*_max_) is the initial amplitude of the Larmor signal when the magnetic field under test is perpendicular to the radial (or axial) direction of the sensor. According to Equation (5), when the magnetic field is perpendicular to the sensor’s radial direction, *θ_B_* = 0° and 90° correspond to the angles between the magnetic field and the coil’s axial and radial magnetic fields, respectively; if the magnetic field is perpendicular to the sensor’s axial direction, the corresponding angular relationships are completely reversed.

The omnidirectionality testing experiment was conducted in the Yichang Weak Magnetic Measurement Laboratory, which is capable of generating a magnetic field with a stable direction and magnitude.

**Figure 18:**
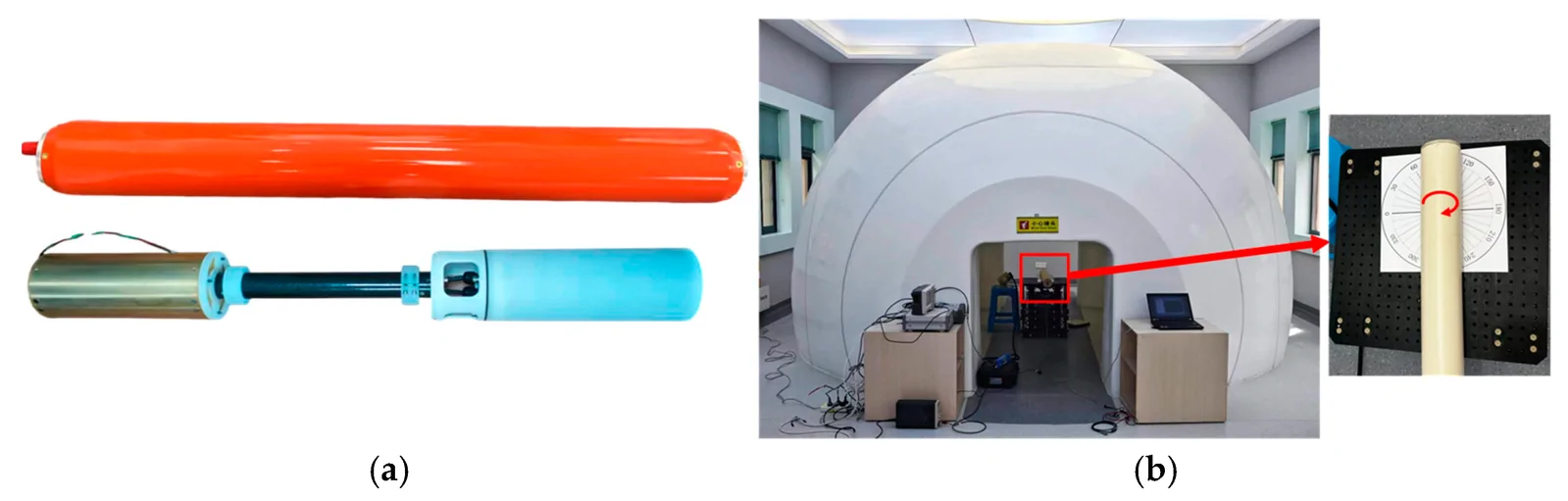
(**a**) The marine base station OVM equipped with the omnidirectional low-frequency coil. (**b**) The omnidirectionality testing of the marine base station magnetometer.

**Figure 19:**
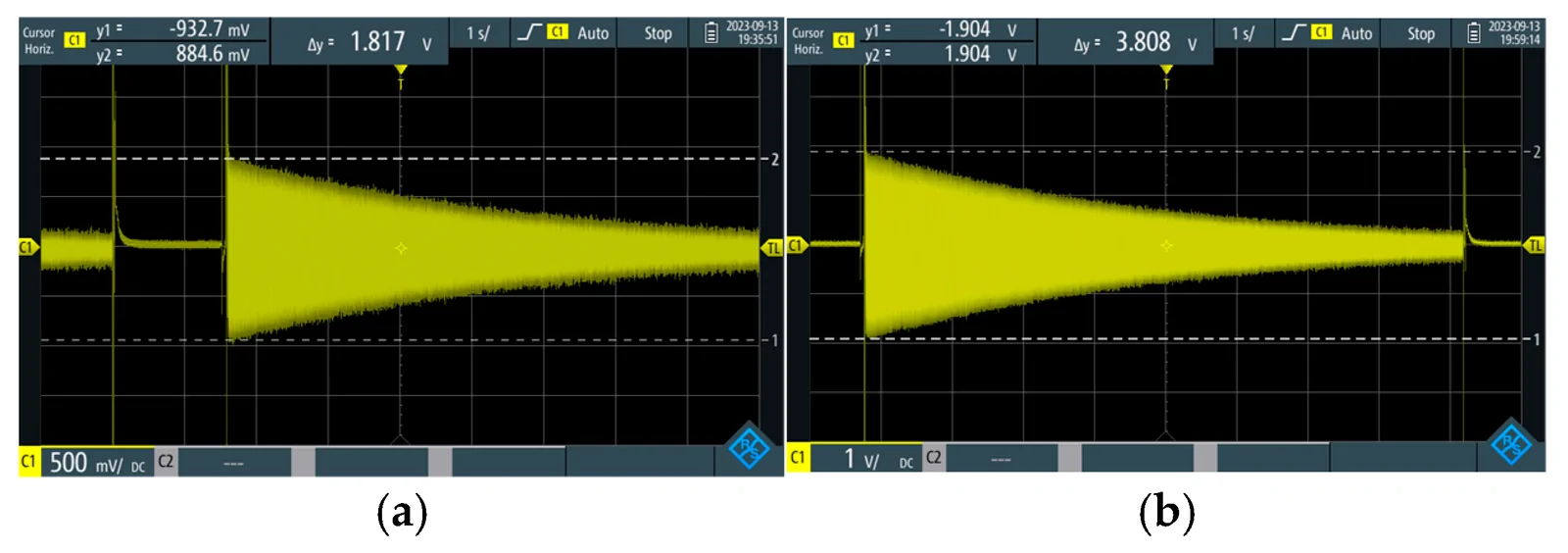
(**a**) The Larmor signal when the sensor axis is parallel to the magnetic field. (**b**) The Larmor signal when the sensor axis is perpendicular to the magnetic field.

As shown in Figure 18a, the upper orange cylinder is a glass fiber pressure-resistant chamber, which can withstand water pressure at a depth of 1000 m. The lower part illustrates the internal structure of the device, mainly composed of an Overhauser sensor, an electronic chamber and a connecting rod. In Figure 18b, the sensor is placed at the center of a uniform magnetic field emitter (white hemispherical structure), which can provide a uniform magnetic field ranging from 20 μT to 120 μT, covering the entire geomagnetic field spectrum. In this study, the magnetic field strength for the measurement was set to 60,000 nT. Its omnidirectional performance is evaluated by rotating the sensor while varying its axial orientation. Figure 19 presents the Larmor signals measured with the sensor axis aligned parallel and perpendicular to the magnetic field, which are 1.817 V and 3.808 V, respectively.

**Figure 20:**
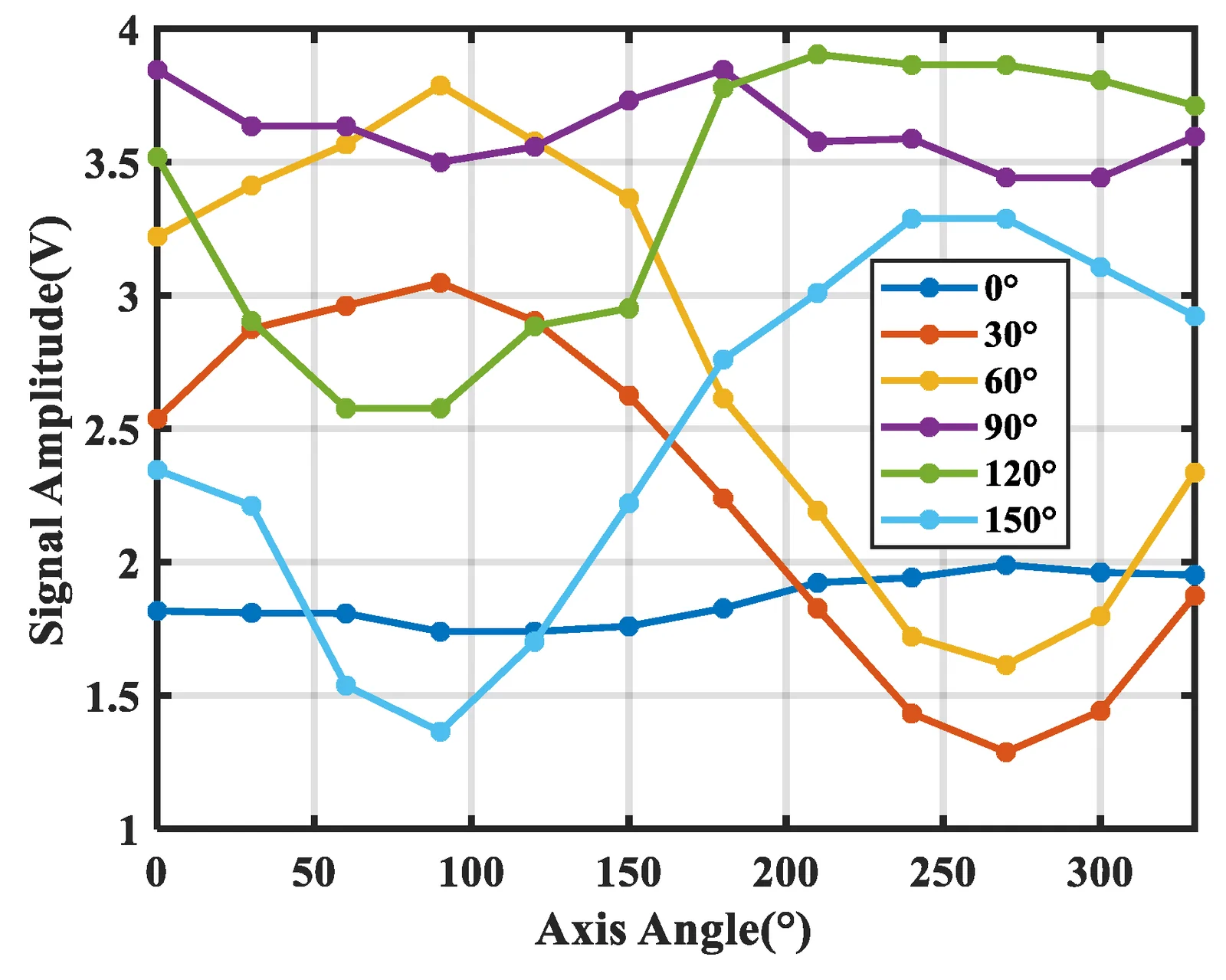
The initial amplitude of the Larmor signal (peak-to-peak value) at different angles.

To comprehensively evaluate the omnidirectional characteristics of the sensor, the following experiments were conducted in this study. Under controlled conditions, while maintaining a constant angle between the sensor axis and the external magnetic field vector, the dependence of the initial Larmor signal amplitude on the axial rotation angle (axis angle) was investigated by executing a full-range rotation of 0–330° around the sensor axis with an angular increment of 30°. On this basis, to systematically explore the modulation laws of the spatial geometric topology on the signal amplitude, the initial relative angle between the sensor axis and the magnetic field was further varied. Consequently, six sets of independent measurements were performed across a range of 0–150° (in steps of 30°), ultimately yielding the six characteristic curves depicted in Figure 20.

As shown in Figure 20, most experimental groups (e.g., 30°, 60°, 120°, and 150°) exhibit distinct quasi-periodic sinusoidal fluctuations in signal amplitude with respect to the axial angle, accompanied by clear phase shifts between different configurations. Conversely, the two anomalous curves (0°and 90°) demonstrate excellent angle insensitivity (i.e., omnidirectional stability) across the full range of angles, making them highly suitable for measurement environments sensitive to attitude perturbations. Specifically, the 90° configuration group (purple curve) consistently maintains a high-level amplitude between 3.4 V and 3.8 V throughout the entire axial angle range. This configuration yields the highest global signal gain, serving as the optimal operating point for pursuing a high signal-to-noise ratio (SNR). Crucially, across all tested configurations and the entire range of axial angles, the Larmor signal amplitude of the system remains strictly greater than 1.0 V, indicating the complete absence of dead zones.

To quantitatively evaluate the omnidirectional coefficient of the sensor, this study calculated the ratio based on the sum of the initial amplitudes of the two characteristic curves at 0° and 90°. The results demonstrate that the experimentally measured omnidirectional coefficient is 0.477, which is in strong agreement with the theoretical calculated value of 0.485. The slight discrepancy of 0.008 is primarily attributed to manufacturing tolerances during the coil winding process and minor magnetic interferences in the testing environment. Although the physical experimental validation focused on the final optimized sensor, the theoretical comparison shown in Figure 17 clearly confirms that the addition of compensation coils significantly enhances the radial magnetic field components, effectively validating the proposed design.

### 4.2. Sensitivity Estimation

Sensitivity is an important indicator of a magnetometer, reflecting the instrument’s relative uncertainty in repeated measurements under a stable magnetic field. The magnetic field *B* measured by the magnetometer usually consists of the reference magnetic field *B_e_* and four forms of random noise: diurnal variation interference *B*_d_, temperature drift noise *B*_L_, external high-frequency electromagnetic interference noise *B*_H_, and the magnetometer’s inherent reading noise *B*_R_. Thus, the magnetic field *B* measured by the magnetometer can be expressed as [25]

*B* = *B*_e_ + *B*_d_ + *B*_L_ + *B*_H_ + *B*_R_ (34)

If the measured magnetic field *B* only contains the reference magnetic field *B*_e_ and the inherent reading noise *B*_R_ of the instrument, the standard deviation (STD) can be adopted to evaluate the sensor sensitivity:

(35) $$\mathrm{STD}=\sqrt{\frac{1}{n-1}{\sum_{i=1}^{i=n}\left(B_{i}-\bar{B}\right)}^{2}}$$

where *B*_i_ is the magnetic field value measured by the magnetometer and *n* is the number of magnetic fields.

The fourth-order difference function, which has high-pass characteristics, can filter out the diurnal variation interference *B*_d_ and low-frequency noise *B*_L_ in the magnetic field. The equation for the fourth-order difference is as follows:

*D*_i_ = *B*_i−2_ − 4*B*_i−1_ + 6*B*_i_ − 4*B*_i+1_ + *B*_i+2_ (36)

where *D*_i_ is the fourth-order difference value of the magnetic field value *B*_i_.

The sensitivity of the magnetometer can be represented by the root mean square (RMS) of the fourth-order difference values:

(37) $$\mathrm{Sen}=\frac{1}{\sqrt{70}}\sqrt{\frac{1}{N-1}{\sum_{i=1}^{i=N}\left(D_{i}-\bar{D}\right)}^{2}}$$

where *N* is the number of *D_i_* and $\bar{D}$ is the average value of *D_i_*.

**Figure 21:**
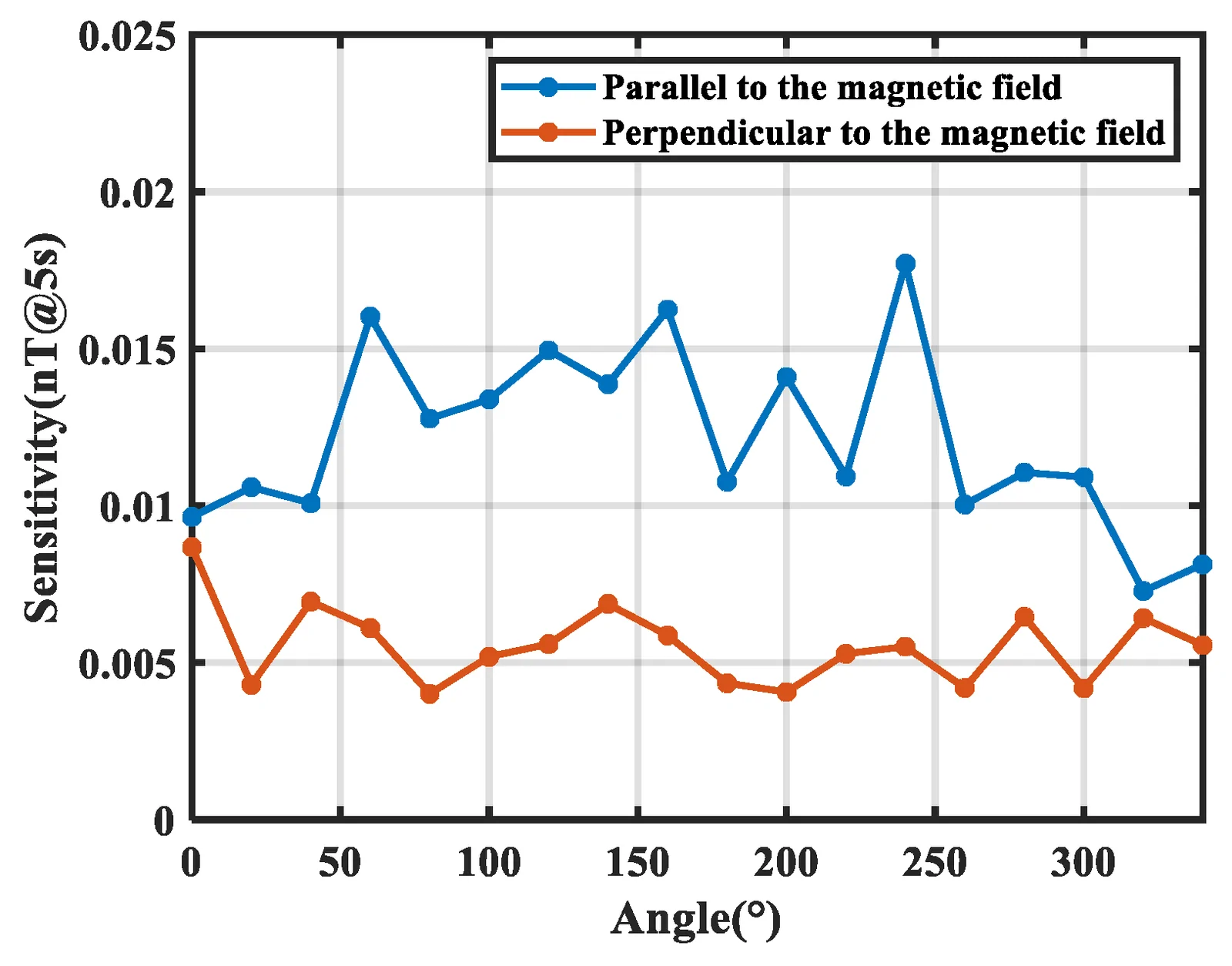
Results of the omnidirectional sensitivity test.

In the magnetic field shown in Figure 18b, diurnal variation interference *B*_d_ and external high-frequency electromagnetic interference noise *B*_H_ are negligible. The fourth-order difference method can effectively eliminate the temperature drift noise *B*_L_, making it an ideal approach to evaluate the magnetometer sensitivity. The experiment was carried out in the weak magnetic laboratory shown in Figure 18b. The sensor axis of the magnetometer was aligned parallel and perpendicular to the magnetic field. The sensor was rotated 360° around its own axis, and data were continuously collected for 5 min at each orientation. The fourth-order difference method was used to calculate the sensitivity at different rotation angles for the two installation states. The results are shown in Figure 21 below.

When the sensor was positioned perpendicular to the magnetic field, the calculated sensitivity ranged from a minimum of 0.004 nT@5s (sensitivity with a cycling rate of 5 s) to a maximum of 0.009 nT@5s. When the sensor was aligned parallel to the magnetic field, the angle between the polarization field and the magnetic field decreased, resulting in a reduction in the Larmor signal. However, the noise level remained unchanged, leading to a lower signal-to-noise ratio. Consequently, sensitivity increased compared to the perpendicular orientation, with a minimum of 0.007 nT@5s and a maximum of 0.018 nT@5s.

## 5. Conclusions

The omnidirectionality of the Overhauser magnetometer sensor is determined by the uniformity of the polarizing magnetic field in two orthogonal directions. An omnidirectional coefficient is proposed to characterize two orthogonal magnetic field components, where an ideal omnidirectional sensor with a coefficient of 1 maintains an unchanged Larmor signal amplitude and sensitivity regardless of orientation. By optimizing the coil structure and parameters according to the calculated omnidirectional coefficient, an eight-segment inclined polarizing coil system with compensation coils was designed with a theoretical omnidirectional coefficient of 0.485. In an artificial magnetic field environment, the omnidirectional coefficient was measured to be 0.477, which is in high agreement with the theoretical result. Furthermore, sensitivity evaluations under extreme conditions demonstrated high-performance stability regardless of sensor attitude. Overall, the proposed omnidirectional coefficient in this paper is suitable for quantitative optimization of polarizing coil structures and parameters, and it is beneficial for improving design efficiency in order to enhance sensor omnidirectionality.

## Data Availability

The original contributions presented in this study are included in the article. Further inquiries can be directed to the corresponding authors.
